# The role of autophagy in the progression of HIV infected cardiomyopathy

**DOI:** 10.3389/fcell.2024.1372573

**Published:** 2024-07-17

**Authors:** Yuting Sun, Mengmeng Xu, Qinchun Duan, Joseph L. Bryant, Xuehong Xu

**Affiliations:** ^1^ Laboratory of Cell Biology, Genetics and Developmental Biology, College of Life Sciences and University Hospital, Shaanxi Normal University, Xi’an, China; ^2^ Zhejiang Cancer Hospital, Hangzhou Institute of Medicine (HIM), Chinese Academy of Sciences, Hangzhou, Zhejiang, China; ^3^ Department of Pediatrics, Morgan Stanley Children’s Hospital, Columbia University, New York, NY, United States; ^4^ Institute of Human Virology, University of Maryland School of Medicine, Baltimore, MD, United States

**Keywords:** cardiomyopathy, highly active antiretroviral therapy (HAART), human immunodeficiency virus (HIV), acquired immunodeficiency syndrome (AIDS), autophagy, mitochondria, mTOR, rapamycin

## Abstract

Although highly active antiretroviral therapy (HAART) has changed infection with human immunodeficiency virus (HIV) from a diagnosis with imminent mortality to a chronic illness, HIV positive patients who do not develop acquired immunodeficiency syndrome (AIDs) still suffer from a high rate of cardiac dysfunction and fibrosis. Regardless of viral load and CD count, HIV-associated cardiomyopathy (HIVAC) still causes a high rate of mortality and morbidity amongst HIV patients. While this is a well characterized clinical phenomena, the molecular mechanism of HIVAC is not well understood. In this review, we consolidate, analyze, and discuss current research on the intersection between autophagy and HIVAC. Multiple studies have linked dysregulation in various regulators and functional components of autophagy to HIV infection regardless of mode of viral entry, i.e., coronary, cardiac chamber, or pericardial space. HIV proteins, including negative regulatory factor (Nef), glycoprotein 120 (gp120), and transactivator (Tat), have been shown to interact with type II microtubule-associated protein-1 β light chain (LC3-II), Rubiquitin, SQSTM1/p62, Rab7, autophagy-specific gene 7 (ATG7), and lysosomal-associated membrane protein 1 (LAMP1), all molecules critical to normal autophagy. HIV infection can also induce dysregulation of mitochondrial bioenergetics by altering production and equilibrium of adenosine triphosphate (ATP), mitochondrial reactive oxygen species (ROS), and calcium. These changes alter mitochondrial mass and morphology, which normally trigger autophagy to clear away dysfunctional organelles. However, with HIV infection also triggering autophagy dysfunction, these abnormal mitochondria accumulate and contribute to myocardial dysfunction. Likewise, use of HAART, azidothymidine and Abacavir, have been shown to induce cardiac dysfunction and fibrosis by inducing abnormal autophagy during antiretroviral therapy. Conversely, studies have shown that increasing autophagy can reduce the accumulation of dysfunctional mitochondria and restore cardiomyocyte function. Interestingly, Rapamycin, a mammalian target of rapamycin (mTOR) inhibitor, has also been shown to reduce HIV-induced cytotoxicity by regulating autophagy-related proteins, making it a non-antiviral agent with the potential to treat HIVAC. In this review, we synthesize these findings to provide a better understanding of the role autophagy plays in HIVAC and discuss the potential pharmacologic targets unveiled by this research.

## Introduction

By preventing the development of acquired immunodeficiency syndrome (AIDs), highly active antiretroviral therapy (HAART) has changed the landscape of illness for patients infected with human immunodeficiency virus (HIV) from a universally fatal diagnosis into a manageable chronic illness. As patients with HIV are living longer, other aspects of HIV infection have come into the forefront. For instance, even patients with good viral load and a relatively healthy immune system often suffer from a high rate of HIV-associated cardiomyopathy (HIVAC) (Pinto and da Silva, 2018; Feinstein, 2023; Freiberg, 2023). Prior to the widespread use of HAART, a high proportion of patients with AIDS had cardiac abnormalities (Pinto and da Silva, 2018; Sinha and Feinstein, 2020) and AIDs patients with cardiac abnormalities had a 5.86 hazard ratio for death in comparison to patients with idiopathic cardiomyopathy (Felker et al., 2000). Despite HAART, the prognosis for HIV infected patients with cardiomyopathy remains similarly grim (Freiberg, 2023). A meta-analysis assessing cardiac function in 2,242 asymptomatic patients with well controlled HIV-1 demonstrated systolic dysfunction in 8.3% of patients and diastolic dysfunction in 43.4% (Cerrato et al., 2013). HIVAC disease now accounts for a quarter of deaths in patients with HIV and is associated with 4.0 and 6.5 times higher risk for death, in asymptomatic and symptomatic patients, respectively (Crum et al., 2006). Reduced myocardial systolic function in HIVAC patients was correlated with high lipid and fibrosis indexes in the myocardial tissue (Thiara et al., 2015). This holds true in non-human primate models as Rhesus macaque monkeys infected with HIV-like simian immunodeficiency virus (SIV) showed HIVAC-like dilated cardiomyopathy and fibrosis correlating with elevated numbers of myocardial CD163+ macrophages (Shannon et al., 2000; Walker et al., 2014). Despite the clinically well-characterized nature of HIVAC, prognosis for these patients remains poor due to a lack of treatments directly targeting the mechanisms behind HIVAC development (Ntsekhe and Baker, 2023).

The drivers behind HIVAC development is assumed to be multifactorial. Factors believed to contribute to the development of HIVAC include heightened cytokines expression, co-infection with bacteria or viruses known to affect the myocardium, HIV induced autoimmunity, and, most recently, direct infection of the myocardium by the HIV virus (Anand et al., 2008; Otis et al., 2008; Sillman et al., 2008; Meng et al., 2013; Sinha and Feinstein, 2020; Perkins et al., 2023). Here we discuss research suggesting HIV infection induced dysregulation of myocardial autophagy a mechanism for HIVAC development. These studies present autophagy modulators as potential pharmaceutical targets for normalizing autophagy function and improving cardiac function in HIV patients.

## Multi-entry of bloodstream HIV into heart in HIV patient

HIV-related cardiomyopathy involves multiple structures in the heart, including the coronaries, pericardium, endocardium, myocardium, and valvular structures as well as indirect involvement on preload and afterload through pulmonary and systemic vasculature (Manga et al., 2017; So-Armah et al., 2020). In a large population study of patients who died from acquired immunodeficiency syndrome AIDS, cardiac disease was documented in 18.6% (82 of 440) of patients and an additional 2.7% (12/440) who suffered from dilated cardiomyopathy and 6.8% who suffered from (30/440) lymphocytic interstitial myocarditis. Of the 82 patients with cardiac disease, 33.3% (28/84) had infective endocarditis, 63.1% (53/84) had pericardial effusion, and 1.2% of patients (3/84) had myocardial sarcoma and lymphoma (Barbaro et al., 1998). Of the 29 patients with active infective endocarditis, *in situ* hybridization with HIV specific probes showed active HIV myocarditis in 25 of these patients (Barbaro et al., 1998).

A 2017 study reported HIV infection of cardiac endothelial cells leads to an interleukin-1/-6 and tumor necrosis factor-alpha stimulated release of pro-inflammatory cytokines that ultimately resulted in chronic endovascular inflammation and subsequent life-threatening endocarditis and cardiac dysfunction (Monsuez et al., 2007; Duprez et al., 2012; Manga et al., 2017). Structurally speaking, an echocardiographic study on 803 HIV-positive patients found 77% of patients to have valvular dysfunction (Reinsch et al., 2013; Hu et al., 2023). In animal models, CCR5 and CXCR4 receptor independent HIV cardiomyocyte infection has been demonstrated to have a profound impact on cardiomyocyte function. Scanning electron microscopy showed invasion through this process as mediated by NRVM gp120 and adjacent perivascular CD68-positive macrophages (alkaline phosphatase-BCIP) resulted in cardiomyocyte damage and subsequently caused cardiac dysfunction in neonatal rat ventricular myocytes (NRVM) (Fiala et al., 2004; Fiala et al., 2004; Herskovitz and Gendelman, 2019; Lodge et al., 2020).

**FIGURE 1:**
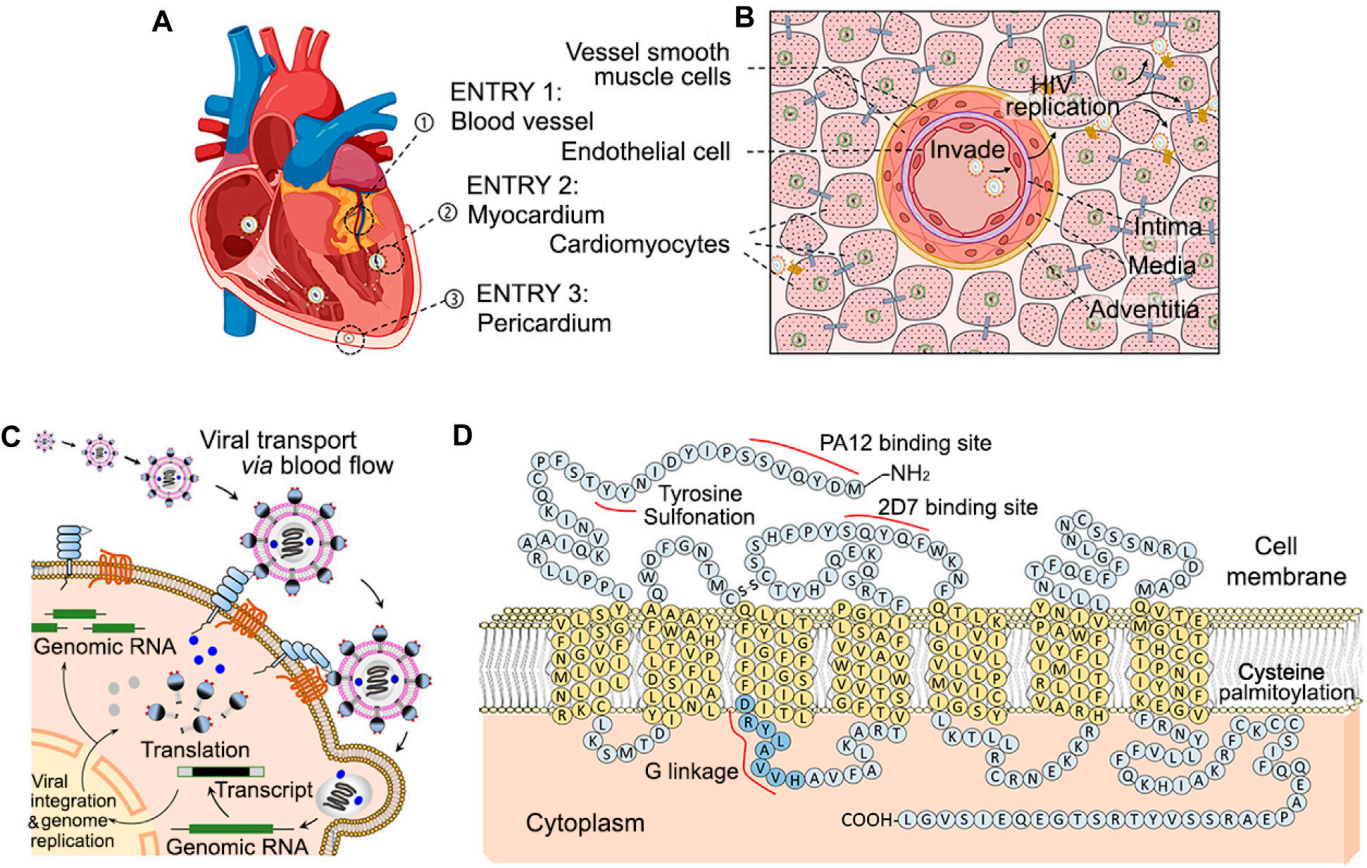
Diagram of multiple entry sites of circulating HIV into cardiomyocytes, and cellular mechanism of CCR-5 mediated invasion. In HIV infected patients, systemically circulating HIV are able to enter and infect cardiomyocytes through three paths [Panel **(A)**]. Bloodborne HIV particles can invade through endothelial cells of the coronary arteries (➀), directly affect cardiomyocytes through the endocardium of atrial and ventricle chMTambers (➁), and reach pericardial cardiomyocytes from the pericardial space (➂). HIV infection via the coronaries require penetrance through the coronary intima, media and adventitia and thus accompanying HIV infection HIV of the coronaries themselves [Panel **(B)**]. Circulating HIV invades cardiac cells such as endothelial cell and cardiomyocytes via HIV GP-120 binding to cell surface CD4 and CCR5 associated to binding sites of PA12 and 2D7 [Panel **(C, D)**].

Despite the clear involvement of HIV in all three layers of the heart, the exact location through which HIV viral particles initiate cardiac invasion remains unclear. There are three avenues through which cardiac HIV infection could occur: coronary invasion, endothelial invasion via cardiac chambers, and pericardial invasion. HIV in systemic circulation eventually reach the coronary arteries through which viral particles can infect the endothelium, intima, media, and adventitia and eventually the myocardium. HIV viral particles in circulation also come into direct contact with the cardiac chamber endothelium, leading to lethal inflammation endocarditis prior to infection of the cardiomyocytes (Figures 1A, B). While traveling along the bloodstream, HIV particles are transported into heart and can directly infect endothelial cells and/or cardiomyocytes through the above three pathways. These viral particles anchor onto cell membranes via interaction of HIV GP-120 to cytoplasmic integrated protein CD4 and CCR5. Viral genomic RNA is then injected into the cells and proceed to hijack host genomic DNA with HIV reverse transcriptase (Figures 1C, D), which begins the production process of millions more particles. Lastly, pericardial distribution of HIV can lead to significant inflammation and pericardial effusion over the visceral layer of the epicardium (Wang et al., 2020).

HIV can cause a variety of cardiac disease via one or more of above three possible paths of entry. In the following review, we focus on the mechanistic role autophagy plays in HIV infection regardless of avenue of entry into cardiomyocytes and discuss possible pharmaceutical targets against autophagy dysregulation in HIVAC and HAART therapy.

## Autophagy flux in human HIV diseases

Autophagy is a cellular process integral to normal cellular regulation, recycling, and decomposition of old, unnecessary, damaged or dysfunctional intracellular components as well as external pathogens. This natural process undergoes four phases—cytoplasmic autophagosome formation, fusion of autophagosome and lysosome, degradation of dysfunctional component, and utilization of repurposed amino acids [Refer to Reviews: Bagherniya et al., 2018; Kaur and Debnath, 2015; Miyamoto 2019]. More than 50 different autophagy-related (ATG) proteins are involved in the autophagy pathway, and ATG dysfunction is critical to the development many diseases including cancer, neuronal disorders, liver disease, diabetes, and heart diseases [Refer to Reviews: Klionsky et al., 2021; Levine and Kroemer, 2019; Ramesh et al., 2019]. However, the link between autophagy and HIV associated heart disease has yet to be explored. In this review, we focus on the role of autophagy dysregulation in HIV associated cardiac pathology and explore how recent characterization of autophagy can potentially be harnessed to provide treatments benefiting HIV patients.

Autophagy has been shown to play a critical a role in HIV-1 related complications. As a function vital to normal cellular catabolism, autophagy is not only vital for eliminating cellular waste, intracellular pathogens, and damaged organelles, but also needed for cellular synthesis and maintaining energy for cell renewal. Together these functions act as an intracellular recycling system that facilitate dynamic circulation of catabolized building blocks, thereby promoting biosynthesis, boosting ATP production, and maintaining nutrient homeostasis (Mizushima and Komatsu, 2011; Chiramel et al., 2013; Kaur and Debnath, 2015; Ramesh et al., 2019; Chen et al., 2023). Several recent *in vitro* and *in vivo* investigations have unveiled the fundamental role of autophagy in system specific manifestations of HIV. For example, a significant increase of autophagy markers Beclin1, autophagy-specific gene 5 (Atg-5), Atg-7, and LC3-II were observed in the brain tissue of HIV-1 encephalitis patients. This suggests autophagy dysregulation may play an important role in the pathogenesis of other devastating neurological complications of AIDS (Levine and Sodora, 2006; Mizushima, 2020; Judith and Berlioz-Torrent, 2023).

As a cellular function long considered a major regulator in cardiac homeostasis and function, autophagy is a key suspect in HIVAC. Autophagy loss-of-function models with constitutional deletion of atg5 and beclin 1 (Nakai et al., 2007) develop severe cardiac pathology. Similarly, cardiac-specific deletion of autophagy gene forkhead box O1 (FoxO1) (Hariharan et al., 2010; Popov et al., 2023) also develop severe cardiac defects. Cardiac dysfunction in both models is due to accumulation of misfolded proteins and dysfunctional organelles caused by autophagy dysfunction. Protein-aggregation mouse models generated by activating major suppressors of autophagy, such as mammalian Ste20-like kinase 1 (Mst1) and mammalian target of rapamycin (mTOR), also exhibit severe cardiac disorder.

Importantly, removing accumulated misfolded proteins by re-activating the autophagy pathway can recover dysfunctional mitochondria and repair damaged DNA, which in turn, rescue cardiac function (Maejima et al., 2013; Sciarretta et al., 2018a; Sciarretta et al., 2018b; Gatica et al., 2022). Studies have also shown autophagy activation to inhibit chronic ischemic remodeling and mediate cardiac adaptation to overload stress by reducing the accumulation of misfolded proteins such as amyloid, pre-amyloid oligomers, and poly-ubiquitin proteins (McLendon and Robbins, 2015; Gupta et al., 2017; Levine and Kroemer, 2019). Autophagy is now considered a cellular process vital to healthy cardiomyocyte aging, protein quality control, and pathogenesis (Levine and Kroemer, 2008; Wang et al., 2008; Shirakabe et al., 2016; Sciarretta et al., 2018b; Klionsky et al., 2021). As increasing evidence mounts of HIV directly disrupting its regulators and proteins, autophagy is under investigation as a key component in HIVAC pathogenesis.

## HIV infection disrupts autophagy flux and leads to HIVAC development

**FIGURE 2:**
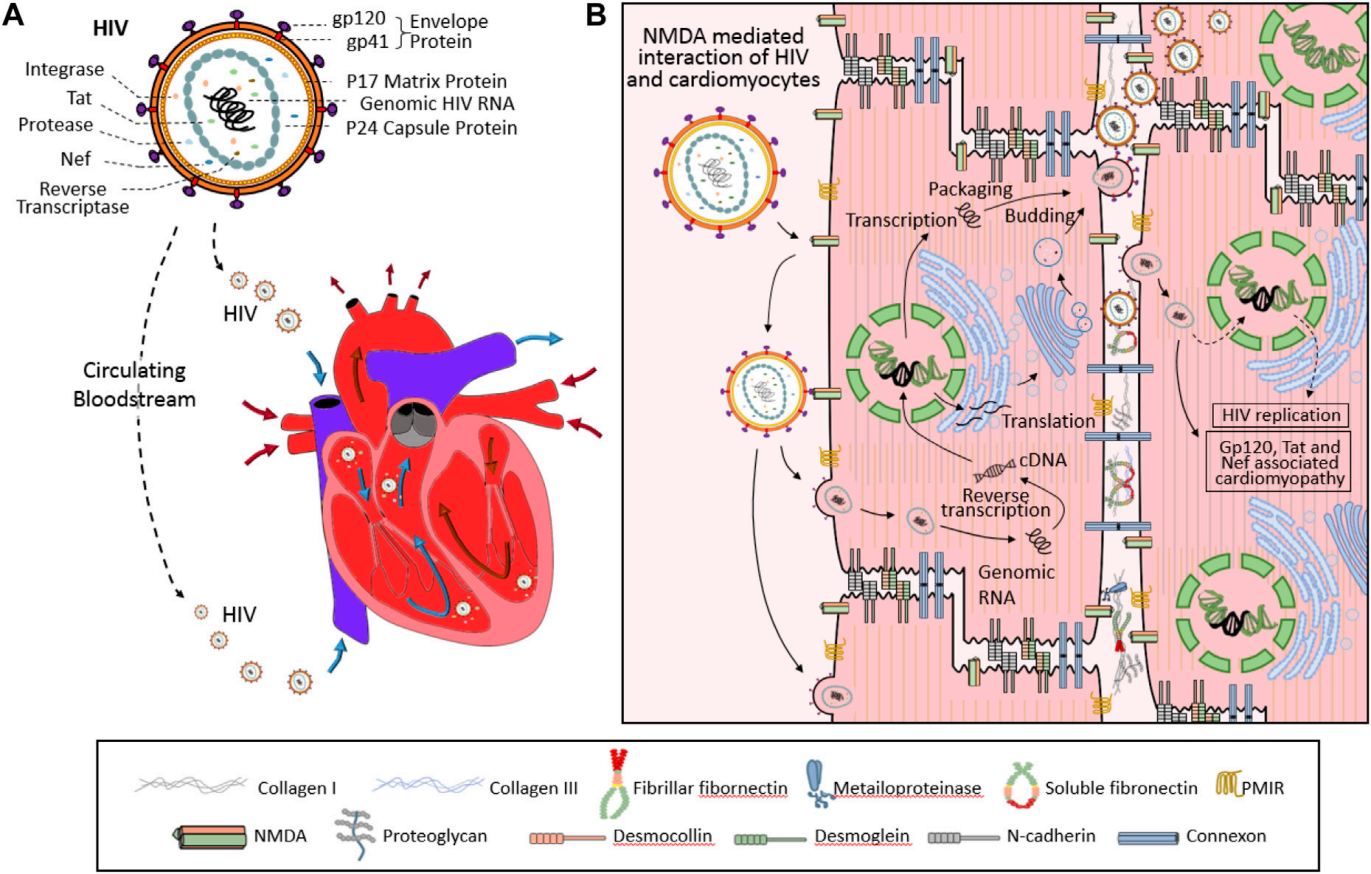
Diagram of the infective process of human immunodeficiency virus (HIV) in cardiomyocytes. Once within the blood stream, the HIV (upper in Panel **(A)**) virus enters the heart via the superior and inferior vena cava (lower in Panel **(A)**) then binds to NMDA on the surface of the myocardium. Interaction between viral gp120 and cellular CD4/CCR5 or membrane integrated receptor (PMIR) allows the virus to enter the cell. After this, the viral replication process begins as it integrates viral RNA into host genomic DNA (Panel **(B)**) and hijacks biological processes inherent to cardiomyocyte function.

**FIGURE 3:**
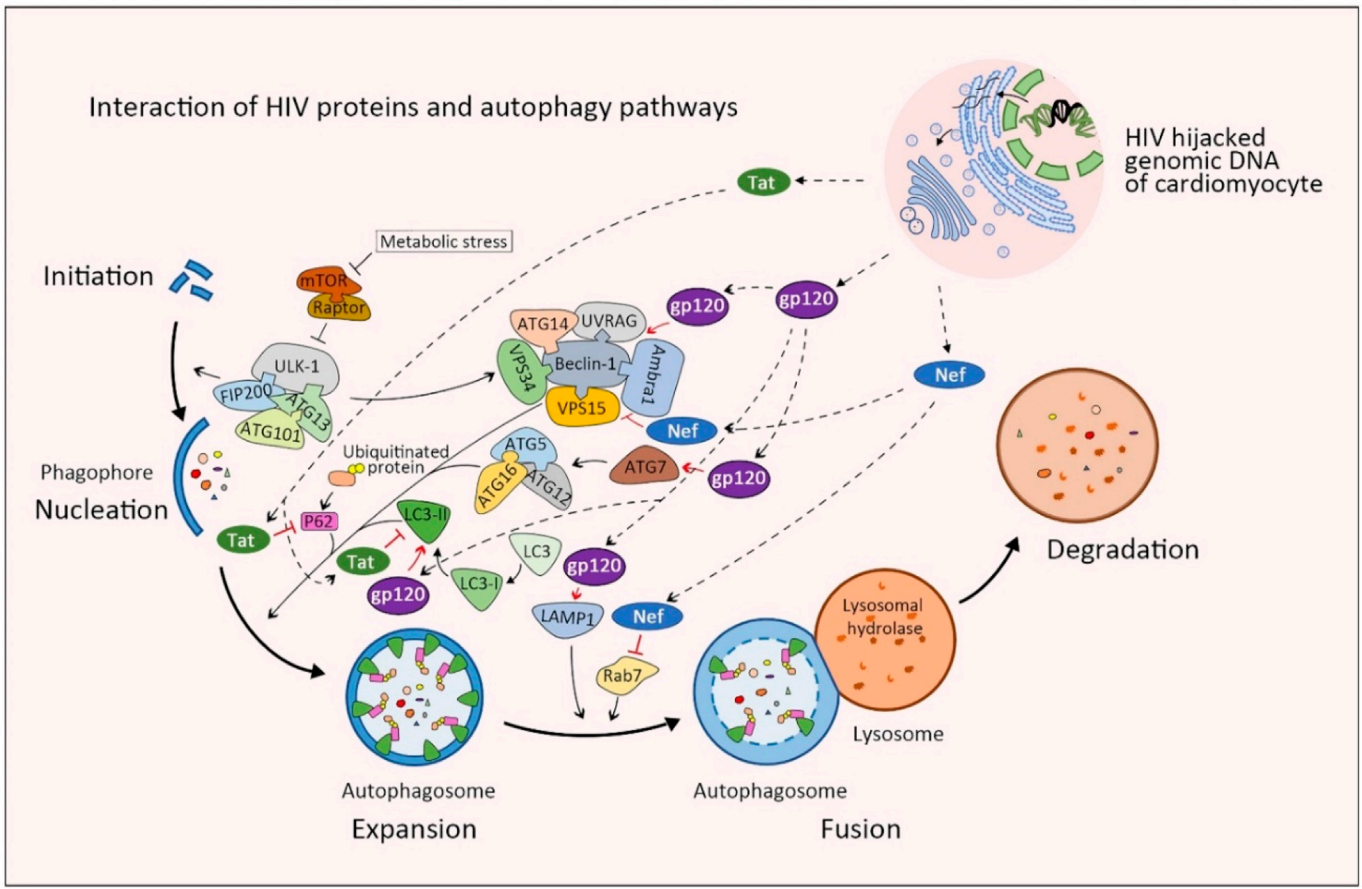
Schematic of protein interactions between normal autophagy and HIV proteins in cardiomyocytes. Viral Tat blocks autophagy by degrading autophagic proteins including SQSTM1/p62 and LC3-II, which halts the autophagosome at the expansion phase. Conversely, viral gp120 promotes the expansion and maturation phases of autophagosome by enhancing the expression of ATG7, Beclin 1, LAMP1 and LC3-II. Finally, viral Nef targets Beclin-1 and Rab7 to inhibit autophagy at the fusion phase. The combination of these changes in autophagy results in an expanded number of autophagosomes that cannot fuse to degrade dysfunctional proteins. Movement of viral factors are indicated with broken-line arrows. Blunt-head arrows (┴) indicate inhibition; Open-head arrows (↓) indicate promotion; Red lines (−) indicate action by viral proteins on cellular proteins; Black lines (−) indicate action by cellular proteins on other cellular proteins.

HIV-1 infection has recently been shown both in animal models and patients to affect basal cardiomyocyte autophagy flux. HIV-1 infected cardiomyocytes express significantly higher levels of type I and II microtubule-associated protein-1 β light chain (LC3), which has been shown to inhibit autophagy in cardiomyocytes after long-term culture. Subsequent cells cultured within media collected from of HIV-1-infected cardiomyocyte cultures also resulted in the accumulation of LC3-II, ubiquitin, and SQSTM1/p62 labeled pre-maturation and degradation autophagosomes (Gupta et al., 2017; Zhao et al., 2021). This accumulation of autophagosomes within HIV-1 infected cardiomyocytes in addition to the classic mechanisms of HIV infection: hijacked reverse transcription, integration of viral RNA into host genomic DNA, and subsequent change in cellular activity, likely all contribute to the development of HIVAC (Figure 2). In addition to the inherent damage caused by the loss of conserved cellular catabolism, dysfunctional autophagy also contributes to myocardial superinfection susceptibility (Ramesh et al., 2019). Here we summarize the molecular mechanisms through which HIV-1 negative regulatory factor (Nef), glycoprotein 120 (gp120), and transactivator (Tat) interact with proteins vital to phases of the autophagy cycle and analyze their roles in HIVAC disease progression (Figure 3).

### HIV-1 infection is dependent on and amplified by hijacked autophagy pathways

Autophagy’s key role in initial HIV invasion and genomic integration is well-characterized. HIV-1 envelope (Env) components activate the mTOR pathway in dendritic cells, which serves to suppress dendritic cell autophagy and allow infection transfer to CD4^+^ T cells (Blanchet et al., 2010; Caucheteux et al., 2023). Increasing evidence now suggest autophagy also plays a key role in optimizing HIV-1 replication and viral particle production (Killian, 2012; Campbell and Spector, 2021). Using HeLa-derived TZM-bl cells transfected with small interfering RNA (siRNA) specific against the IIIB strain of HIV-1, Brass et al. found key regulators of autophagy specific genes (ATG7, ATG8, ATG12, ATG16L2) and lysosomal-associated genes (CLN3 and LapTM5) to be indispensable for HIV-1 replication (Brass et al., 2008). Furthermore, RNAi-mediated knockdown of autophagy factors PIK3R4, ATG4A, ATG5, and ATG16 in SupT1 cells inhibited HIV-1 replication (Eekels et al., 2012). Using VSV-Gpseudotyped NL4-3 HIV-1 in U937 cells (Kyei et al., 2009) similarly showed HIV-1 Gag precursors can be co-localized, copurified, and co-precipitated with autophagy-regulating LC3-II to facilitate the processing of Gag into structural virion core proteins (Lennemann and Coyne, 2015; Campbell and Spector, 2021). Importantly, while this process amplifies virus production via promotion of autophagosome expansion, viral Nef simultaneously inhibits the autophagosome maturation that destroys HIV virions (Figure 3). The ability of HIV to highjack autophagy flux for enhanced viral replication within host cells is key to its success as a pathogen. As a major engine of cellular homeostasis with pathways conserved across cell types, autophagy dysregulation is a likely mechanism through which HIV can directly induce cardiac dysfunction.

## Viral nef inhibits autophagy in cardiomyocytes

The multifunctional viral accessory protein Nef plays a role in viral replication, assembly, budding, infection, and immune evasion—all vital to the life span of the virus (Mailler et al., 2019). This 27–32 KDa protein is expressed from early on in the infection process and is key in many aspects of viral infection and CD4^+^ T-cell suppression. Nef downregulates CD4 and major histocompatibility complex-I (MHC-I) expression in infected cells, thereby allowing infected cells to avoid detection by cytotoxic T-cells. HIV Nef secreted in exomes are also thought to trigger CD4^+^ T cell apoptosis, thereby contributing to the hallmark CD4 lymphopenia seen in AIDS (Lenassi et al., 2010; Caucheteux et al., 2023). HIV-1 Nef is also known preserve newly produced infectious viruses by constraining autophagosome maturation and acidification (Chiramel et al., 2013; Zhao et al., 2021; Kyei et al., 2009). Nef inhibits autophagosome maturation by increasing the expression of LC3-II and interacting with Beclin-1 to reduce autophagic degradation of the viral capsid protein p24. In addition to these key infective and immunosuppressive activities, recent studies have shown Nef to pay a role in HIVAC through interruption of autophagy. Guptal et al. reported cardiac specific expression of Nef to inhibit cardiomyocyte autophagy, leading to an accumulation of dysfunctional protein that interfered with myocardial contractile function and ultimately led to significant *in vitro* cardiomyocyte toxicity and death. They showed that HIV Nef accumulating in exosomes mature into autophagosomes by interacting with viral maturation factors, Beclin-1 and Rab7, subsequently causing substantial storage of poly-ubiquitin and p62 proteins in HIV-1 infected cardiomyocytes (Gupta et al., 2017; Cheney et al., 2020). Removal of functional poly-ubiquitin and p62 proteins then halt autophagosome at the maturation stage, leading to an accumulation of dysfunctional autophagosomes in cardiomyocytes (Figure 3). Interestingly, even during adequate HAART therapy, viral Nef has been found to accumulate in HIV and SIV infected cardiomyocytes, which may explain why HIVAC is prevalent even when the infection is considered clinically well-controlled with normal CD4 counts and low viral load.

## Viral Gp120 induces autophagy in cardiomyocytes

As a key protein on the surface of HIV, gp120 mediates invasion of HIV and SIV viruses into host cells. During the initial infection, viral gp120 first binds to CD4 on the host-cell surface, leading to a change in its spatial conformation which in turn mediates the interaction of viral glycoprotein 41 (gp41) with host C-C Chemokine Receptor type 5 (CCR5) (Xu, 2020). This process triggers the fusion of cellular and viral membranes, releasing viral RNA into the host cytoplasm (Saayman et al., 2015). In addition to this key function, HIV-1 gp120 has been shown to induce autophagy in neurons and T lymphocytes. This phenomenon has since also been identified in vitro H9c2 cardiomyocytes. Recent *in vitro* studies have further traced this affect to HIV-1 gp120 induced expression of ATG7, Beclin 1, LAMP1 and LC3-II—autophagy related proteins found in cardiomyocytes (Meng et al., 2013). This study also found interaction between gp120 and N-methyl-D-aspartate receptor (NMDA) to be a requirement for gp120 induced autophagy in H9c2 rat cardiomyocyte cells and can be significantly inhibited with NMDA receptor inhibitors (Meng et al., 2013). Gp120 was further proven to act through the autophagy pathway as inhibitors of autophagy regulators, c-Jun N-terminal kinase (JNK) and phosphoinositide 3-kinase (PI3K), rescue gp120-induced autophagy hyper-activation in H9c2 rat cardiomyocytes. Other *in vivo* work has since verified the direct inhibitory action of JNK inhibitor (SP600125) and class III PI3K inhibitor (3-MA) on the expression of autophagy regulators ATG7, Beclin one and LAMP1. Importantly, these inhibitors reduced cellular autophagy and improved structure and function of HIV-infected cardiomyocytes (Meng et al., 2013).

## Viral Tat regulates autophagy in cardiomyocytes and neurons

HIV Tat is necessary for integration of viral genomic RNA into host genomic DNA for viral replication and expression (Debaisieux et al., 2012; Jiang et al., 2018). In addition to this already pivotal role, recent research has suggested Tat also plays a vital role in the development of dilated cardiomyopathy in HIV infected individuals. Transgenic mice engineered to express HIV-1 Tat developed reduced percentage of fractional shortening in isolated cardiomyocytes. Physiologically, this resulted in depressed peak left ventricular systolic pressure (LVSP) and elevated left ventricular end-diastolic pressure (LVEDP) consistent with decreased systolic and diastolic function intermediated through cardiac Ryanodine Receptor associated Calcium homeostasis (Raidel et al., 2002; Fang et al., 2009; Alomar et al., 2022). The specific mechanism through which Tat acts on cardiomyocyte autophagy has been traced to autophagy related proteins LC3-II and p62. Tahrir et al. confirmed the ability of HIV-1 Tat to decrease the expression of LC3-II and p62 in cardiomyocytes. This disrupts the formation and maturation of autophagosomes, resulting in cardiomyocytes with an accumulation of misfolded proteins and damaged organelles. This in combination with Tat’s ability to induce oxidative stress in cardiomyocytes significantly impacts cardiomyocyte health and homeostasis (Tahrir et al., 2018).

HIV Tat’s ability to alter cellular homeostasis also occurs in HIV-associated neurodegenerative disease (HAND) (Fields et al., 2015; Mehrbod et al., 2019; Ramesh et al., 2019; Marino et al., 2020). Treatment of B103 neuronal cells and primary mouse neurons with increasing concentrations of recombinant HIV-1 Tat produces dose-dependent inhibition of LC3-II and ubiquitin-binding sequestosome 1 (SQSTM1/p62) protein expression. Furthermore, in immunoprecipitation and double-immunolabeling experiments, HIV Tat was co-localized with lysosomal-associated membrane protein 2A (LAMP2A) in the cytoplasm. This co-localization inhibited autophagosome-lysosome fusion in neurons and caused an accumulation of misfolded proteins and damaged organelles similar to that seen in HIV-1 infected cardiomyocytes (Fields et al., 2015). *In vivo* models of HAND confirmed the physiologic effect of these findings, when neurons from glial fibrillary acidic protein- Tat (GFAP-Tat) mice showed altered LC3-II levels and increased autophagosome accumulation correlating with neurodegeneration. Notably, these effects were reversed by treatment with rapamycin, an autophagy activating mTOR inhibitor (Fields et al., 2015). HIV Tat expression has also been shown to induce dose-dependent higher levels of BCL associated athanogene 3 (BAG3) induced autophagy in glioblastoma cell lines (Bruno et al., 2014). Interestingly, BAG3 is known to interact with molecular chaperone heat shock protein 70 (HSP70) to modulate cellular autophagy in the heart (Inomata et al., 2018). Recent work in C57BL/6J mice has also linked SQSTM1/p62 expression, which is downregulated in HANDs, with cardio-protectively during stress-generating short-term exercise (Feng et al., 2019; Yamada et al., 2019; Ning et al., 2023). Other rat models have also shown the SQSTM1/p62 associated Nrf2 signaling pathway to play a critical role in autophagy-dependent cardiac protection against myocardial ischemia and age-related cardiac dysfunction (Bhide et al., 2018; Ning et al., 2023). Given these known functions of QSTM1/p62 and Nrf2 in cardiomyocytes and the pathogenic role these same proteins play in HIV-associated neurodegenerative disease, the same proteins likely play a similar role in HIV-associated cardiac disease.

### MTOR inhibitor rapamycin rescues myocyte toxicity by upregulating autophagic flux

MTOR is a critical serine-threonine protein kinase involved in major cellular and physiological functions, such as metabolism and protein synthesis, proliferation and cell growth, and cell death and contractile-autophagy. Through the formation of two functional complexes, i.e., mTORC1 (composed of mTOR, TEL1-2, RAPTOR and mLST8) and mTORC2 (mTOR, TEL2, PROTOR1-2, mSin1, RICTOR and mLST8), mTOR plays an important role in cardiac development and physiological function. The mTOR complex composed of mTOR and regulatory protein raptor or rictor plus other binding proteins, regulates ribosomal biogenesis and protein synthesis via he ribosomal protein S6 or the translation initiating factor eIF4E [Refer to Reviews: Konopka et al., 2023; Quarles et al., 2020; Sanches-Silva et al., 2020]. Inhibition of mTOR with rapamycin can attenuate pathological cardiac hypertrophy and improve the function of aging heart via inhibition of the cardiac proteasome activity. Via rapamycin blockade of mTORC1, pAMPK is allowed to interact with Atg13/ULK1/FIP200 form the autophagosome (Refer to Review: Sciarretta et al., 2012). As a negative regulator of chaperone-mediated autophagy (CMA) and macroautophagy, mTORC2 can direct the PDGF-induced phosphorylation of Ser473 of Akt and eventually affects autophagy by targeting on Ser234 and Ser295 phosphorylated Beclin-1/VDAC1/GFAP (Refer to Review: Ballesteros Alvarez et al., 2021). Since mTORC1 or/and mTORC2 regulated the pathway via different proteins, treatment strategies targeting autophagy in HIVAC via the mTOR pathway need to be selective for pAMPK or AKT signaling.

MTOR signaling is considered a central regulator of autophagy and is known to regulate many aspects of autophagy in HIVAC (Sciarretta et al., 2018a). Due to the important role autophagy plays in cellular health, the mTOR signaling pathway is key in regulating cell growth, proliferation, and longevity (Sciarretta et al., 2018a; Sanches-Silva et al., 2020). Previous research has shown inhibition of mTOR to have beneficial effects against cardiomyopathy, atherosclerosis, cardiac hypertrophy, and heart failure (Akbay et al., 2020; Sanches-Silva et al., 2020). Recent research linking autophagy pathways to HIV and HAART associated CVD has unveiled the mTOR pathway as a source of possible molecular targets against the pathogenesis of CVD in HIV patients (Das et al., 2014; Campbell et al., 2018; Ogunbayo et al., 2018; Crater et al., 2022; Singh et al., 2022).

### MTOR inhibition, caloric restriction, and the calcium related PI3K/mTOR/BRD4 pathway as pharmacologic pathways for autophagy mediated cardiac disease

Improving autophagy flux has been shown to improve cardiac function in animal models of multiple types of cardiac disease. As pathologies driven by inhibited autophagy flux, HIV and HAART associated cardiomyopathy are likely to respond to treatments promoting autophagy. As previously discussed, the mTOR inhibitor rapamycin has proven to be effective cross a wide spectrum of CVD diseases driven by autophagy (Wohlgemuth et al., 2007; Dai et al., 2014; Linton et al., 2015; Miyamoto, 2019; Quarles et al., 2020). Rapamycin treatment also reversed age-dependent cardiac hypertrophy and diastolic dysfunction in multiple mouse studies (Flynn et al., 2013; Dai et al., 2014; Konopka et al., 2023). Similar to the mechanisms found in other CVD, rapamycin treatment altered the expression of genes involved in calcium regulation, mitochondrial metabolism, hypertrophy, and inflammation in age related cardiac dysfunction (Flynn et al., 2013; Konopka et al., 2023). Given the abundance of evidence supporting the ability of rapamycin to improve cardiac function in CVD driven by dysfunctional autophagy, this mTOR inhibitor is likely to be an effective therapy in highly autophagy dysfunction-driven HIV and HAART associated cardiac disease.

Another therapy comparable to rapamycin for improving autophagy-driven, age-related cardiac dysfunction was short-term caloric restriction (Dai et al., 2014; Escobar et al., 2019). Caloric restriction is well known as a promotor of cellular autophagy through the mTOR pathway (Dai et al., 2014; Bagherniya et al., 2018; Mannick and Lamming, 2023; Shi and Collins, 2023). A recent study using mice models of age-dependent cardiac hypertrophy showed the proteome half-lives of old hearts significantly increased after 10 weeks of short-term caloric restriction or rapamycin treatment (Dai et al., 2014). This was accompanied by substantially reduced p62 and Beclin-1 levels and an attenuation of age-dependent protein oxidative damage and ubiquitination. These changes boosted mitochondrial function, ATP production, the citric acid cycle, and fatty acid metabolism as well as decreased the prevalence of proteins involved in glycolysis and oxidative stress response. The reduction in protein damage and ubiquitination contributed to increasing half-lives of exhausted proteins in cardiomyocytes and subsequently reversed pre-existing cardiac dysfunction in caloric restricted and rapamycin treated mice (Dai et al., 2014). A study using longer term caloric restriction showed enhanced autophagy and protected cardiac contractile function in 24-month old mice after 20 weeks of caloric restriction. This protective effect was also found to act via increased expression of autophagy proteins LC3-II and Beclin-1 (Han et al., 2012; Maldonado et al., 2022).

Amplifying autophagy outside of the myocardium may also be protective against HIVAC. Hulsmans et al. demonstrated that resident macrophages concentrated in the mouse atrioventricular node regulate the electrophysiological activity of these cardiomyocytes (Hulsmans et al., 2017; Zhou et al., 2018). A pilot study has shown macrophage health to affect cardiac electrical conduction and promote cardiac regeneration (Hulsmans et al., 2017). Thus HIV infection of macrophages likely results in changes in cardiomyocyte electrophysiology and contributes to arrhythmia in HIVAC. HIV infection alter macrophage activity by inhibiting autophagosomes formation and maturation in macrophages. This process is mediated by the phosphoinositide 3-kinases (PI3Ks)/mTOR/bromodomain 4 (BRD4) pathway (Campbell et al., 2018; van Montfort et al., 2019; Tao and Drexler, 2020). Recent investigations have shown PI3K/mTOR inhibitor dactolisib (NVP-BEZ235) and PI3K/mTOR/BRD4 inhibitor SF2523 to be effective at inhibiting HIV-1 replication within macrophages (Campbell et al., 2018; van Montfort et al., 2019; Tao and Drexler, 2020). Given the convergence of these inhibitors at the autophagy regulating mTOR signaling pathway, pharmacological inhibition of macrophage autophagy may assist in preventing macrophage facilitated HIVAC.

## Rapamycin promotes autophagy across multiple pathologic processes

Given the essential role mTOR plays in vital cellular functions, this pathway and its substrates have been studied across many cell types. Many of these studies have found pharmacologic inhibition of this pathway to be beneficial for a variety of pathogenic processes. A common thread across these studies is that mTOR inhibition promotes autophagy to promote clearing of misfolded or dysfunctional proteins, organelles, and other cellular components (Nair and Ren, 2012; Kumar et al., 2020; Liu and Sabatini, 2020; Taylor et al., 2020). For instance, mTOR inhibitor rapamycin reduced renal podocyte injury and improved the glomerular filtration barrier in rat models for renal injury by increasing autophagy levels in the podocytes of treated animals (Wu et al., 2013). Rapamycin also promoted cell death in human neruoblastoma cell lines by arresting them at the G0/G1 phase of the cell cycle. Evaluation of these rapamycin treated, arrested cells showed increased number of autophagosomes and elevated expression of autophagosome proteins Beclin-1 and LC3-II/LC3-I (Lin et al., 2018). Rapamycin induced autophagy is also theorized to provide a protective effect in human neurodegenerative disorders, such as Alzheimer’s disease, Parkinson’s disease, and Huntington’s disease by clearing the key disease feature: aggregate proteins (Rahman and Rhim, 2017; Mputhia et al., 2019; van Montfort et al., 2019).

## Rapamycin treatment normalizes autophagy flux in HIV-infected cardiomyopathy

Rapamycin has provided beneficial effects in a range of animal models for cardiovascular disease including rat models of tetrachloride carbon (CCl4) induced cirrhotic cardiomyopathy (Saeedi Saravi et al., 2016), rat models of uremic cardiomyopathy (Haller et al., 2016), mouse models of genetic hypertrophic cardiomyopathy (Xu et al., 2014), and mouse models of metabolically driven CVD (Reifsnyder et al., 2018; Singh et al., 2022). Across all of these models, an increase in autophagy was observed followed by an improvement in myocyte contractility or cardiac function. Rapamycin was also reported to provide a cardio-protective effect in an animal model for myocardial ischemia reperfusion injury (Khan et al., 2006). In the reperfusion injury study, rapamycin treated mice had 64% decreased area of necrosis and apoptosis in comparison to the untreated group (Khan et al., 2006; Shimada et al., 2021; Xie et al., 2021). Rapamycin also prevented cardiac dysfunction in models of chronic disease. Treatment with rapamycin restored fractional shortening and ejection fraction in mouse models of diabetes mellitus (T2D) by blocking enhanced phosphorylation of mTOR, reducing oxidative stress, and normalizing glucose metabolism regulators (Das et al., 2014). While these studies modeled disease with very different pathogeneses, treatment with rapamycin altered autophagy or mitophagy to improve cardiac function across all models.

As a cardiac disease with underlying pathologies in mitochondrial regulation and reduced autophagy, HIV-associated cardiomyopathy is expected to respond to mTOR inhibitor normalization of mitophagy and autophagy. As previously discussed, rapamycin treatment has been shown to counteract various single components of HIV infection. The mTOR inhibitor increased cellular autophagy in Nef-expressing NRVCs, to normalized cellular apoptotic body formation and facilitate the redistribution and removal of aggregated autophagic proteins LC-II, p62, Beclin1, ubiquitin, and Rab7. This reduced Nef-induced cytotoxicity by allowing cardiomyocytes to normalize autophagy flux. A process that was aided by the simultaneous redistribution of transcription factor EB (TFEB) and cytoplasmic lysosome contents (Gupta et al., 2017). Given the close relationship between mTOR pathway subjects, autophagy, and mitochondrial homeostasis, to the development of HIVAC, a direct inhibition of this aberrant pathway would be an optimal strategy for effective treatment of HIVAC.

### Complications of targeting the autophagy pathway HIV-Infected patients with cardiomyopathy

As described above, the autophagy pathway is regulated by mTOR in the cardiac pathology of HIV-infected patients. Although results from animal models demonstrate rapamycin treatment can stabilize autophagy flux and maintain mitochondrial homeostasis to prevent worsening cardiomyopathy (Gupta et al., 2017) and its positive effects have been shown in many cardiac diseases such as cirrhotic cardiomyopathy (Saeedi Saravi et al., 2016), uremic cardiomyopathy (Haller et al., 2016), genetic hypertrophic cardiomyopathy (Xu et al., 2014), atherosclerosis, and heart failure (Akbay et al., 2020; Sanches-Silva et al., 2020). Rapamycin is not targets to cardiomyocytes alone, and thus use is not without risks. A special risk consideration in the setting of HIV infected patients is rapamycin’s immune modulating effects via antigen-specific lymphocytes, and innate immune cells such as dendritic cells and macrophages (Janes and Fruman, 2009). Given that even minor immune-suppression with use of the drug could have a large impact on viral load in HIV-infected patients, the risk and benefit of this drug must be carefully studied and weighed. Specifically, testing of this drug in HIV infected animal models would be vitally important.

## HIV induces dysregulation of cardiomyocyte homeostasis via mitophagy

As the cellular energy farm, healthy mitochondria is critical to cardiomyocyte function. Normal selective degradation of defective mitochondria through mitophagy is critical to maintaining a healthy mitochondrial population. When mitochondria are damaged fewer ATP are produced to power cardiomyocyte contractility. Damaged mitochondria also generate dangerous amounts of reactive oxygen species (ROS). ROS induced oxidative stress and acidification of the cellular environment causes abnormal post-translational modifications that leads to a catastrophic feedforward cycle of oxidative damage. This environment decreases ATP production by altering the expression of peroxisome proliferator-activated receptors (PPARs), decreasing the activity of tricarboxylic acid cycle (TCA) cycle enzymes, disrupting the electron transport chain (ETC), and further changing oxidative phosphorylation. Alterations of metabolic enzyme activity and ATP production eventually trigger heart failure (HF) as cardiomyocytes lack the ATP necessary to survive (Parihar and Parihar, 2017; Avula et al., 2021; Bulló et al., 2021). Thus, as a regulator of cellular mitochondrial homeostasis, mitophagy is a crucial factor in cardiomyocyte function and cardiac health (Morales et al., 2020; Abdel-Rahman et al., 2021; Ning et al., 2023).

### Mitochondria and calcium homeostasis

Many calcium signaling modulators including inositol trisphosphate receptors (IP3R), ryanodine receptors (RyRs), and calcium-sensing receptors (CaSR) are regulated by mitochondria. Perturbations in these calcium mitochondrial relationships are well known to be related to abnormal cell death. Mitochondrial collapse can lead to calcium release and apoptosis, while conversely, the binding and ubiquitylation of IP3R3 by F-box protein FBXL2 can also limit mitochondrial calcium influx to promote apoptosis (Pan et al., 2004; Kuchay et al., 2017; Zhang et al., 2022). Given the role of mitochondria in cellular survival, it is not surprising that mitochondrial abnormalities leading to aberrant calcium signaling have also been identified in cardiovascular disease (Wang et al., 2018; Xu et al., 2018; Li et al., 2022; Wang et al., 2022), cancer (Shaikh et al., 2016; Xu et al., 2018; Garbincius and Elrod, 2022), and HIVAC (Xu, 2020). Recent studies in rat cardiomyocytes have also found expression of HIV Tat to significantly reduce mitochondrial calcium uniporter (MCU)-mediated mitochondrial calcium uptake, which dampens electrophysiological activity in cardiomyocytes. As cardiac contractility in highly dependent on calcium signaling, any changes in calcium homeostasis can induce cardiac dysfunction. Thus, disruptions in mitochondrial housekeeping by mitophagy can affect cardiomyocyte calcium homeostasis and function and lead to clinical cardiac dysfunction.

### HIV-1 Tat induced mitochondrial oxidative stress and abnormal mitophagy in cardiomyocytes

In addition to affecting cardiomyocyte calcium homeostasis through mitophagy, HIV interaction with mitophagy also disrupts the balance of mitochondrial oxidative stress. Using neonatal rat ventricular cardiomyocytes (NRVC), Tahrir and colleagues characterized the effect of HIV-1 Tat expression on amplifying mitochondrial oxidative stress. Their study found HIV-1 Tat expression to generate an acidic environment by reducing ATP production and oxidative phosphorylation. This is accomplished by directly affecting multiple complexes in the mitochondrial electron transfer chain. HIV-1 Tat decreases levels of complex I subunit NADH dehydrogenase (ubiquinone) 1α subcomplex 4-like 2 (NDUFA4L2) and cytochrome c (complex III) while simultaneously stimulating complex IV Cytochrome C oxidase (Cox-2). These changes serve to downregulate ATP production and upregulate the creation of mitochondrial reactive oxygen species (mROS) (Bertero and Maack, 2018; Tahrir et al., 2018). The accumulation of mROS in NTVCs expressing HIV-1 Tat leads to mutations of mitochondrial DNA (mtDNA), which permanently damage the mitochondrial respiratory chain (Rodríguez-Mora et al., 2015; Tahrir et al., 2018). Such an overproduction of mROS has been characterized as a pathophysiological symptom of cardiomyopathy in many chronic cardiac diseases, such as atherosclerosis, hypertension, and congestive heart failure (Côté, 2005; Sugamura and Keaney, 2011; Koczor et al., 2015; Bertero and Maack, 2018; Li et al., 2022). Furthermore, expression of HIV Tat in NRVCs significantly reduced MCU-mediated mitochondrial Ca2+ uptake, which further affects electrophysiological activity in cardiomyocytes to impact cardiac contractility. Surprisingly, the same study also found HIV associated Tat to trigger hypoxic conditions, which leads to increased mitochondrial mass and altered morphology *in vivo* (Tahrir et al., 2018). These findings suggest HIV Tat’s ability to disrupt the electron transport chain and increase mROS is further amplified by its simultaneous interruption of normal mitophagy checkpoints for dysfunctional mitochondria.

### HAART induces cardiomyopathy by disturbing mitochondrial homeostasis

Despite the improvement in mortality since the advent of HAART, patient with well controlled HIV infection still suffer from high rates of cardiovascular disease (CVD). While this high rate of cardiac disease is attributed to HAART toxicities, the exact mechanism remains largely unknown. Interestingly, recent data has shown that at least one HAART drug, nucleoside reverse transcriptase inhibitor (NRTI) azidothymidine (AZT), causes cardiomyopathy through the same autophagy pathways targeted by HIV infection (Table 1) (Nomura et al., 2017; Lin et al., 2019; Cheney et al., 2021).

**TABLE 1:** Instinctive linking between HIV proteins/anti-AIDs drugs and autophagy in cardiomyocytes.

HIV proteins/Drugs		Viral process	Molecular mechanism	Potential inhibitors or strategies	Cellular effect	References
HIV Proteins	Nef	Participates in virus replication, assembly, budding, infection, and immune evasion	Interacts with Beclin-1 and Rab7	Chemical 35 disrupting Beclin-1 by binding to Bcl-2; TRAF6 triggering MIH autophagy through Rab7 ubiquitination	Increasing cellular toxicity, decreases cellular viability and significant cell death	Gupta et al. (2017), Levine and Kroemer. (2019), Ma et al. (2023), Pan et al. (2023), Zhao et al. (2021)
	gp120	Mediates initial invasion of virus into host cells	Interacts with NMDA receptor to induce the expression of ATG7, Beclin-1, LAMP1 and LC3-II	Possible chemicals targeting on LAMP-1/-2 autophagosome formation involved with TMEM175 channel activity controlling lysosomal pH	Causing cell destruction	Mehrbod et al. (2019), Meng et al. (2013), Ning et al. (2023), Pan et al. (2023), Saayman et al. (2015), Zhang et al. (2023)
	Tat	Enhances initiation of virus replication and promote transcription and translation of mRNA	Degradation of SQSTM1/p62 and LC3-II	DC-LC3in-D5 inhibiting autophagy by attenuating LC3B lipidation, reducing autophagic formation and substrate degradation	Destroying homeostasis, increased mitochondrial mass and altered mitochondrial morphology	Alomar et al. (2022), Fan et al. (2021), Jiang et al. (2021), Ma et al. (2019), Tahrir et al. (2018)
HAART Drugs	Zidovudine (AZT)	Combines with viral DNA polymerase to inhibit replication of virus	Induction step in autophagosome accumulation	A nucleoside analog reverse-transcriptase inhibitor used against the HIV virus	Inducing accumulation of dysfunctional mitochondria and ROS production	Hsue and Waters. (2019), Lin et al. (2019)
	Ritonavir (RTV)	Suspends HIV particles in an immature, non-infective state	Activation of platelet activity and increases the level of TGF-β1	A pharmacokinetic enhancer to boost the activity of other HIV medicines	Contributing to cardiac dysfunction and fibrosis	Alomar et al. (2022), Hsue and Waters. (2019), Laurence et al. (2018)
	Abacavir	Inhibits the activity of HIV reverse transcriptase and prevents the replication of viral DNA	Activates platelets and aggregates platelet-TGF-β1	A nucleoside reverse transcriptase inhibitor, a guanosine analog, a carbocyclic 2′-deoxyguanosine	Contributing to myocardial fibrosis and dysfunction	Alomar et al. (2022), Hsue and Waters. (2019), Laurence et al. (2018)

NMR, Nuclear magnetic resonance (NMR) spectroscopy; MIH, autophagy, Mycobacterium-infected host autophagy.

In an *in vivo* study using immortalized mouse myoblast cell line C2C12, AZT and its active metabolite, AZT-triphosphate (AZT-TP), was confirmed to cause cell death by altering mitophagy flux and over-promoting mROS production. In this study, AZT treated C2C12 cells demonstrated a high index of mitochondrial mass and ROS production as measured with MTR Red staining and CM-H2DCFDA fluorescence, respectively. Furthermore, green fluorescent protein labeled LC3 (GFP-LC3) tracking, Western blotting, flow cytometry, confocal microscopy, and electron microscopy all confirmed AZT to have a significant dose- and time-dependent inhibitory effect on late stage autophagosome maturation. This AZT inhibition of late autophagy causes an accumulation of dysfunctional mitochondrial with hyperpolarized membranes and increased mROS. To further isolate mitophagy as the culprit for AZT generated changes, the changes were replicated by pharmacological and genetic inhibition of cardiomyocyte autophagy. Most importantly, these changes were rescued by pharmacological stimulation of autophagolysosomal biogenesis by mTOR inhibitors (Lin et al., 2019; Akbay et al., 2020). These results support the importance of mitochondrial homeostasis in the development of cardiomyopathy and present a new mechanism for HAART induced cardiomyopathy that may explain the continued clinical prevalence of HIVAC in HIV patients despite appropriate antiviral therapy.

The parallels between cardiomyopathy caused by HIV infection and HAART highlights the importance of their shared pathogenesis: mitophagy dysregulation. In both instances, mitochondrial health is compromised by increased mROS and acidification, which in turn leads to abnormally large and dysmorphic mitochondria. Finally, HIV and AZT also inhibit late autophagy, causing an accumulation of dysfunctional mitochondrial that eventually results in cellular death. Most importantly, these shared changes in mitophagy are reversible when treated with an autophagy stimulating mTOR inhibitor.

## HAART-INDUCED cardiac dysfunction and fibrosis is mediated by platelet-derived TGF-Β1 and can be suppressed by autophagy

In addition to the role autophagy plays in mitochondria-dysfunction driven HIVAC, autophagy also regulates platelet-derived transforming growth factor beta 1 (TGF-β1) mediated HAART induced CVD (Laurence et al., 2018; Madzime et al., 2021). Like NRTI AZT, which disturbed mitochondrial homeostasis, protease inhibitors (PIs) can also induce ROS overproduction, ubiquitin-proteasome system (UPS) dysregulation, and functional alteration of ion channels in the myocardium (Reyskens and Essop, 2014; Alvi et al., 2018). Although this effect does not appear to act directly on the mitochondria, PIs cause disruption of excitation–contraction coupling, induced calcium overload, and endoplasmic reticulum stress. These changes indirectly alter mitochondrial respiration and impair ATP generation. The PI Ritonavir (RTV) has also been shown to induce an accumulation of ubiquitinated proteins and cause an alteration in lipid metabolism (Reyskens and Essop, 2014; Alvi et al., 2018; Hsue and Waters, 2019). These changes all correlate with a range of clinical presentations of clinical presentations associated with CVD including: ECG abnormalities, lower left ventricular ejection fractions (LVEF), higher pulmonary artery pressure (PASP), dyslipidemia, diabetes, and coronary artery disease (CAD) (Alvi et al., 2018; Hsue and Waters, 2019; Avula et al., 2021).

A recent study by Laurence et al. has found protease inhibitor RTV induced cardiac dysfunction to be dependent upon the expression level of platelet TGF-β1 (Laurence et al., 2017). In a mouse model treated with physiologic levels of RTV, decline in cardiac output, ejection fraction, and stroke volume was correlated with nearly a 5-fold increase of TGF-β1 in myocyte plasma. Interestingly, this TGF-β1 driven RTV-induced cardiac fibrosis can be markedly reduced by treatment with inhaled carbon monoxide (CO). CO upregulates the inducible heme-oxygenase/endogenous CO pathways, thereby modulating both the canonical (Smad2) and non-canonical (p38) pathways of TGF-β1 signaling. Notably, this process was proven to be autophagy dependent as CO treatment provides no protection against RTV-mediated cardiac fibrosis in autophagy-deficient LC3^−/−^ mice (Laurence et al., 2017). The platelet TGF-β1 dependent pathway for HAART induced CVD is not restricted to PIs. NRTI Abacavir was also found to cause platelet activation and the release of platelet TGF-β1. Identical to PI induced CVD, Abacavir induced activation of platelet TGF-β1 resulted in myocardial fibrosis and subsequent cardiac dysfunction (Satchell et al., 2011; Laurence et al., 2018; Hsue and Waters, 2019).

The convergence of pathogenic mechanisms between both protease inhibitors and nucleoside reverse transcriptase inhibitors presents autophagy as a single, elegantly targetable pathway for the treatment of HAART induced CVD. Given the data discussed above, normalizing autophagy in patients on HAART could recalibrate a range of HAART induced cellular abnormalities. In turn, this improved mitochondrial function, normalized calcium homeostasis, restored ATP production, and stabilized platelet TGF-β1 would prevent the development of HAART induced CVD.

## Parallels between HIVAC and the aging heart—opportunities for pharmacologic intervention

Cardiovascular disease is the number one cause of death in developed nations (Lloyd-Jones et al., 2009). While this collection of diseases is worsened by other factors such as hypertension, dyslipidemia, diabetes, obesity, and smoking, it always accompanies age regardless of lifestyle and risk factors. The key hallmarks of the aging heart are hypertrophy, fibrosis, inflammation and decreased contractility of cardiomyocytes. As a cellular process necessary for clearing away misfolded and dysfunctional proteins, autophagy plays a key role in maintaining cardiomyocyte health and function during the aging progress. Mitophagy, mitochondria specific autophagy, plays an especially important role in the heart as the mitochondria functions to supply ATP for constantly contracting cardiomyocytes and serves as a key regulator in calcium hemostasis. In fact, promoting autophagy has been shown to rescue cardiac disease in some animal models (Khan et al., 2006; Das et al., 2014; Xu et al., 2014; Haller et al., 2016; Saeedi Saravi et al., 2016; Reifsnyder et al., 2018), and has been theorized to decelerate the aging process of the heart (Linton et al., 2015; Shirakabe et al., 2016; Miyamoto, 2019).

## Both autophagy activation and inhibition are key factors in the ageing cardiomyocyte

Similar to HIV infection, aging has been shown to lead to downregulation of autophagy. Rate of autophagy are thought at to first increase with time as autophagy flux is stimulated by increasing levels of oxidative stress and misfolded proteins within the cell. However, this process can only be maintained for a finite amount duration before the stresses exceed a certain intrinsic threshold, after which cells experience a decline and failure in autophagy. This failure ultimately leads to the inhibition of autophagy in time-worn cells (Shirakabe et al., 2016). A recent study unveiled many transcription factors regulating age-dependent autophagy of the heart such as FoxO, TFEB, hypoxia inducible factor-1 (HIF-1), p53, E2F transcription factor 1 (E2F1), nuclear factor-κB (NF-κB), Kruppel-like factor 4 (KLF4), and zinc finger with KRAB and SCAN domains 3 (ZKSCAN3) (Shirakabe et al., 2016). Many cellular autophagy inhibitors found to be active in aging cells have also been described, including mTOR (Sciarretta et al., 2012; Popov et al., 2023) and Mst1 (Hariharan et al., 2010) or inhibition of main autophagy activators, including sestrin2 (Quanet al., 2017; Zahid et al., 2023; Dai et al., 2023), and sirtuin1 (SIRT1) (Hariharan et al., 2010; Morselli et al., 2010). However, the exact mechanism through which cells regulate autophagy and how cells determine the ageing threshold at which an inhibition of autophagy is preferred over activation requires further investigation.

In young healthy hearts, abnormal mitochondria are rapidly removed by mitophagy. However, as autophagy activity decreases with age, mitophagy also decreases (Sun et al., 2015; García-Prat et al., 2016; Abdellatif et al., 2023; Wang et al., 2023), leading to an abnormal mitochondrial membrane and cellular accumulation of dysfunctional mitochondria in the aging heart (Dutta et al., 2012). Excessive ROS produced by aging mitochondria further adds to cellular stress and damages autophagy activation by modifying autophagy related proteins, such as mTOR, Atg3 and Atg7 (Miyamoto, 2019; Kumar et al., 2020; Mizushima, 2020). Blocking autophagy flux increases cellular mROS, which in turn oxidizes RyR2 in HL-1 cardiomyocytes to stimulate the release of calcium, leading to an electrolyte imbalance that promotes cardiac arrhythmia (Murphy et al., 2019; Avula et al., 2021). The PINK1/Parkin pathway has recently been linked to mitochondrial quality control in the ageing mouse, with the recruitment of Parkin to dysfunctional mitochondria obviously weakened in older cardiomyocytes (Hoshino et al., 2013; Li et al., 2023). Interestingly, overexpression of Parkin in the heart increases mitophagy and provides an anti-aging effect that reduces cellular inflammation and improves cardiac function (Hoshino et al., 2013; Leon and Gustafsson, 2016; Miyamoto, 2019; Abdellatif et al., 2023). These findings add Parkin as another potential therapeutic target for normalizing mitophagy in age, HIV, and HAART related CVD.

### Epithelial-to-mesenchymal transition could serve as a possible target against HIV-infected cardiomyopathy

The epithelial-to-mesenchymal transition (EMT) is critical not only for maturing embryonic architecture of organs, but also vitally involved in many adult pathological conditions, wound healing and carcinogenesis (via the TGF-β/Smad2 signaling pathway) (Zhu et al., 2017; Lovisa, 2021; Khamis et al., 2022; Xu XH et al., 2023). In the heart, reversal of EMT in cardiac fibroblasts produces the regeneration of functional vessels through promotion of mesenchymal-to-endothelial transition (MET). This process results in neovascularization and regeneration, leading to decreased cardiac fibrosis, thereby limiting cardiac injury and improving cardiac function (Miyake and Kalluri, 2014; Ubil et al., 2014; Dong et al., 2020; Lovisa, 2021). This process has also been recently shown to be active in HIV associated cardiomyopathy, where manipulation of autophagy-mediated EMT, via the RAGE pathway was shown to improve cardiac fibrosis and cardiac hypertrophy (Zhang et al., 2021; Liang et al., 2022; He et al., 2023). Activation of EMT-like processes were also shown to activate re-differentiation and assist in cardiac regeneration (Aharonov et al., 2020). Thus, given the ubiquity of the process, manipulating MET/EMT also represents a strong potential target for designing therapeutic drugs against HIVAC using the autophagy pathway.

## Conclusion and future perspectives

Despite HAART greatly reducing the morbidity and mortality of HIV and AIDS, patients on appropriate anti-retroviral therapy still experience a much greater rate of cardiovascular disease than the general population, regardless of CD4^+^ count and viral load. This persistent higher risk for CVD has been linked to autophagy dysregulation, which is known to be a key factor in CVD development of a wide range of etiologies. HIV infection leads to intracellular autophagy imbalance through three key viral components, envelope protein gp120, transcriptional activator Tat, and accessory protein Nef. In addition to playing key roles in the viral infection and reproductive process, all three viral components also significantly alter host cell autophagy flux. Gp120 causes over-activation of early autophagy, while Nef and Tat both restrict the final step of autophagosome maturation, resulting in an accumulation of autophagosomes, and dysfunctional proteins and organelle. Tat adds an extra layer of cellular distress by increasing production of mitochondrial ROS, disrupting the mitochondrial electron transport chain necessary for ATP generation, and altering calcium homeostasis. NRTI azidothymidine and PI ritonavir act through similar pathways, leading to a paralleled accumulation of dysfunctional proteins and mitochondria, and increased mROS that leads to HAART induced myocardial dysfunction and fibrosis. Importantly, mTOR inhibitor rapamycin has been shown to normalize autophagy and restore cardiac function in animal models of autophagy-driven cardiomyopathy. Other effectors of autophagy such as carbon monoxide and inhibitors against autophagy proteins SQSTM1/p62, Beclin1, ubiquitin, Rab7, and the PI3K/mTOR/BRD4 pathway have also shown promising cardio-protective effects. Considering the key role autophagy plays and the positive data showing restoring autophagy can improve cardiac dysfunction, normalizing autophagy pathways should be further studied as a treatment of HIV- and HAART-associated cardiomyopathy.
